# Nuclear microRNAs in normal hemopoiesis and cancer

**DOI:** 10.1186/s13045-016-0375-x

**Published:** 2017-01-05

**Authors:** John E.J. Rasko, Justin J.-L. Wong

**Affiliations:** 1 Gene & Stem Cell Therapy Program, Centenary Institute, University of Sydney, Camperdown, 2050 Australia; 2 Sydney Medical School, University of Sydney, Camperdown, NSW 2050 Australia; 3 Cell and Molecular Therapies, Royal Prince Alfred Hospital, Camperdown, 2050 Australia; 4 Gene Regulation in Cancer Laboratory, Centenary Institute, University of Sydney, Camperdown, 2050 Australia; 5 Locked Bag 6, Newtown, NSW 2042 Australia

**Keywords:** miRNAs, Hemopoiesis, Cancer, Gene regulation, Nuclear localization, Blood

## Abstract

Since the discovery of microRNAs (miRNAs) in the early 1990s, these small molecules have been increasingly recognized as key players in the regulation of critical biological processes. They have also been implicated in many diverse human diseases. The canonical function of miRNAs is to target the 3′ untranslated region (3′ UTR) of cytoplasmic messenger RNA to post-transcriptionally regulate mRNA and protein levels. It has now been shown that miRNAs can also bind to the promoter regions of genes or primary miRNA transcripts to regulate gene expression. Such observations have indicated the presence of miRNAs in the nucleus and implied additional non-canonical functions. Nevertheless, the role(s) of nuclear miRNAs in normal hemopoiesis and cancer remains elusive despite a burgeoning literature. Herein, we review current knowledge concerning the abundance and/or functions of nuclear miRNAs during blood cell development and cancer biology. We also discuss ongoing challenges in order to provoke further studies into identifying key roles for nuclear miRNAs in the development of other cell lineages and human cancers.

## Background

Non-coding RNAs are RNA molecules that are not translated into proteins but are nevertheless functional. They include long non-coding RNAs, intronic RNAs, circular RNAs, competing endogenous RNAs, microRNAs (miRNAs) and Piwi-interacting RNAs that are known to regulate gene expression at both the transcriptional and post-transcriptional levels [1–5]. Of all non-coding RNA species, miRNAs are best characterized in terms of their biogenesis and functions.

miRNAs are approximately 22 nucleotides in length. They play major roles in numerous biological processes including cell differentiation, lineage specification, reprogramming, immune response and the cell cycle [6–10]. Almost 30,000 miRNAs in 223 animal and plant species have been annotated in the miRNA database, miRBase (Release 21, June 2014). Even viruses, particularly herpesviruses, encode miRNAs to enhance their replication potential [11, 12]. The biological importance of miRNAs is further highlighted by the deregulation of miRNA expression in many diverse human diseases including cardiovascular [13], neuronal [14, 15], inflammatory [15], dermatological [16], hepatological [17] and malignant diseases [18–20]. Given their key roles in many normal and disease-related processes, it is not surprising that miRNAs are enthusiastically viewed as potential druggable targets. To achieve this goal and anticipate side effects, it is important to understand their mechanisms of action and functions.

It is generally recognized that most mature miRNAs are localized in the cytoplasm along with the four catalytic Argonaute (Ago) proteins, where they contribute to the RNA-induced silencing complex (RISC) [21–23]. It is via this RISC complex that miRNAs regulate gene expression by targeting messenger RNAs (mRNAs). The majority of binding sites for miRNAs are within the 3′ untranslated region (3′ UTR) of target mRNAs in animals, whereas they are often within coding regions in plants [24]. A mature miRNA can bind to the 3′ UTR of a target mRNA based on partial sequence complementarity between the two to initiate one of several mechanisms to reduce mRNA and/or protein levels. These mechanisms include repression of translational elongation, impairment of translation initiation, and decapping and deadenylation of mRNA, which are reviewed extensively elsewhere [25–27].

Notably, numerous nuclear miRNAs have also been reported. These miRNAs may have diverse known or unknown non-canonical functions. Herein, we discuss how nuclear-localized miRNAs, although synthesized through biogenesis pathways identical to cytoplasmic miRNAs, could be shuttled back and retained in the nucleus to exert functions that differ from canonical miRNA actions. We focus especially on our current knowledge concerning the distribution and roles of these miRNAs in hemopoietic and cancer cells.

## Evidence of nuclear-localized miRNAs

Systematic profiling analyses performed on nuclear and cytoplasmic-fractionated RNA samples have concluded that the majority of miRNAs are present in the nucleus [28, 29]. These results indicate that most, if not all, miRNAs have the capacity to shuttle between the nucleus and cytoplasm. This finding is supported by the localization of Ago proteins in the nucleus and a recent discovery that a component of the RISC complexes, TNRC6A, is a nuclear and cytoplasmic shuttling protein that facilitates Ago nuclear transport. [30, 31] In vitro knockdown of Importin 1 and 8 has also been shown to reduce the nuclear localization of Ago proteins and/or miRNAs, indicating that these transporter proteins mediate the translocation of Ago/miRNAs into the nucleus (see reviews by Liang et al*.* [32] and Roberts [33], for details) [32, 33].

In addition to nuclear import mechanisms, there are also miRNA-intrinsic aspects that facilitate nuclear localization. The first evidence was provided by the predominant localization of miR-29b in the nuclei of HeLa and NIH3T3 cells, as directed by a hexanucleotide motif (AGUGUU) at its 3′ terminus [34]. A related miRNA, miR-29a, lacked this hexanucleotide motif and is enriched in the cytoplasm [34]. Notably, a synthetic siRNA harboring the miR-29a sequence engineered to include the AGUGUU motif could be directed into the nucleus, indicating the importance of this motif for nuclear localization [34]. Hwang and co-workers further identified variants of this motif that is associated with the nuclear localization of miRNAs, including UGUGUU, ACUGUU, AGAGUU, AGUCUU, AGUGAU, AGUGUA, AGNGUN [35]. An independent study by Jeffries and co-workers confirmed the presence of these motifs in seven other nuclear-localized miRNAs: miR-30b, miR-30c, miR-19a, miR-374a, miR-374b, miR-590-5p and miR-193b [36].

However, these motifs alone may not be sufficient to direct nuclear localization of miRNAs [28]. For example, miR-92b that possesses a hexanucleotide motif identical to the nuclear-localized miR-29b has been reported to be cytoplasmically enriched [28]. A recent study also failed to identify a relationship between nuclear or cytoplasmic enrichment of miRNAs and their seed sequences, suggesting that seed identity itself is insufficient to determine their predominant localization [36]. As yet unknown sequence-independent factors may exist to retain, detain or reimport specific miRNAs in the nucleus, and they may also be cell-type specific.

**Fig. 1:**
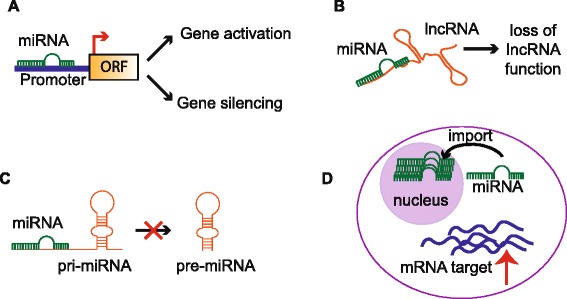
Known roles of nuclear-localized miRNAs. **a** Regulation of gene expression by targeting gene promoters to activate or silence genes. **b** Targeting and suppressing long non-coding RNA (lncRNA) function. **c** Perturbation of miRNA biogenesis via binding to primary miRNA (pri-mRNA) transcripts. **d** Fine-tuning mRNA target expression through detention of miRNAs in the nucleus

Notwithstanding the lack of clarity regarding mechanisms of nuclear miRNA localization, numerous studies have demonstrated their enrichment and functions in myriad mammalian cell types [37–45]. The functions of these nuclear miRNAs include regulation of gene and long non-coding RNA expression [37, 38, 46], controlling the biogenesis of other miRNAs [41] and fine-tuning the expression of mRNA expression in the cytoplasm [43] (Fig. 1). Many of these functions are relevant to hemopoiesis and cancer as outlined in the following sections.

## Nuclear-localized miRNAs in hemopoietic cells

Hemopoiesis is one of the first processes in which the functions of miRNAs were elucidated [47, 48]. Lineage commitment towards blood cell production begins with the hemopoietic stem cell escaping quiescence and the stepwise acquisition of specific myeloid or lymphoid identities [49, 50]. The process involves gradual changes in the expression patterns of hundreds of proteins, including transcription factors [49, 50] and cell cycle regulators [51], many of which are direct or indirect targets of miRNAs.

More than a decade ago, three miRNAs, miR-181a, miR-223 and miR-142 were recognized as key players in myeloid and lymphoid cell differentiation [47]. miR-181a is preferentially expressed in the B-lineage, miR-223 in the myeloid lineage and mir-142 in both B and myeloid lineages [47]. Remarkably, enforced expression of miR-181a led to a doubling of B cells while persistent expression of miR-223 or miR-142 increased the number of T cells by 30–40% [47]. Subsequently, miR-181a has also been found to be crucial for T cell development and function, modulating T cell receptor signalling, in part through its role in the downregulation of multiple phosphatases including SHP2, PTPN22, DUSP5 and DUSP6 [52]. Other confirmed targets of miR-181a include *TCR*, *CD69* and *BCL-2*, all of which are regulated during T cell development [53].

Given the substantial literature on the role of miRNAs in hemopoiesis, studies that determined the cellular localization of miRNAs in hemopoietic cells are relatively few. Most studies have focused on the canonical roles of miRNAs in the post-transcriptional regulation of genes involved in hemopoiesis. The best examples of nuclear miRNA functions have been reported in the context of granulopoiesis (Table 1).

**Table 1:** Nuclear-localized miRNAs in normal hemopoiesis

Nuclear miRNA	Lineage/cell type	Function/activity	Significance	Reference
miR-223	Myeloid	Binds to the promoter region of *NF1A* to induce transcriptional silencing	Repression of *NF1A* to promote granulopoiesis	[42]
miR-706	Myeloid (Lin^−^Sca^+^Kit^+^ hemopoietic stem/progenitor cells, promyelocytes, myelocytes and granulocytes), MPRO, EL4, MEL, A20	Detained in the nucleus to derepress target mRNA (e.g. *Stat1*)	Increased expression of transcription factor (e.g. *Stat1*) to promote granulopoiesis	[43, 57]
miR-690	Myeloid (Lin^−^Sca^+^Kit^+^ hemopoietic stem/progenitor cells, promyelocytes, myelocytes and granulocytes), MPRO, EL4, MEL, A20	Unknown	Unknown	[43]
miR-709	Myeloid (Lin^−^Sca^+^Kit^+^ hemopoietic stem/progenitor cells, promyelocytes, myelocytes and granulocytes), MPRO, EL4, MEL, A20	Unknown	Unknown	[43]
miR-467a*	Lin^−^Sca^+^Kit^+^ hemopoietic stem/progenitor cells, MPRO, EL4, A20	Unknown	Unknown	[43]

The myeloid specific miRNA, miR-223, has been well characterized as playing an essential role in the control of granulopoiesis. In the cytoplasm of myeloid progenitors, miR-223 targets transcription factors MEF2C and NF1A that usually promote myeloid progenitor cell proliferation [54, 55]. The inhibition of cell proliferation in myeloid progenitors coincides with their differentiation into granulocytes. Notably, miR-223 also targets seed sequences in the *NF1A* promoter to induce transcriptional gene silencing via recruitment of the polycomb repressive complex and a consequent increase in DNA methylation levels [42]. These data indicate that both nuclear and cytoplasmic miR-223 work synergistically to silence NF1A during granulopoiesis [42, 54].

Our group has recently reported the enrichment of miRNAs including miR-690, miR-706 and miR-709 in the nucleus of primary Lin^−^Sca^+^Kit^+^ hemopoietic stem/progenitor cells, promyelocytes, myelocytes and granulocytes [43]. We and others have also found miR-690 and/or miR-709 to be nuclear-enriched in various human and mouse cell lines including MPRO, EL4, MEL, A20, L929 and HEK-293T indicating that their functions may be localized to the nucleus [41, 43]. One of these miRNAs, miR-709, has been reported to bind with perfect complementarity to pri-miR-15a and pri-miR-16-1 and inhibit the biogenesis of these miRNAs [41]. This finding has established a previously unanticipated role of nuclear miRNAs in regulating or fine-tuning the expression of other miRNAs.

Our in silico analysis predicted many putative primary miRNA (pri-miRNA) sequences that could be targeted by miR-690, miR-706 or miR-709 with perfect complementarity [43]. Relevant to myelopoiesis, miR-706 is predicted to bind perfectly to a hemopoeitic-specific miR-142-3p, which is known to play an essential role in granulocyte homeostasis and maturation [43, 56]. Nevertheless, we were not able to demonstrate an increase of pri-miR-142-3p processing following inhibition of miR-706 with a hairpin inhibitor against this miRNA [43]. Our result indicates the complexity of factors that determine miRNA binding to its targets in the nucleus, which are not likely to be dependent on sequences alone.

Importantly, we further reported that miRNAs may be retained in the nucleus to fine-tune the expression of mRNA targets [43]. For example, miR-706 enrichment in the nucleus is associated with decreased cytoplasmic miR-706 expression. Consequently, the expression of its targets such as the myeloid transcription factors, *Stat1*, increases to promote myeloid differentiation [43, 57].

## Nuclear-localized miRNAs in cancer

The nuclear localization of miRNAs in cancer cells is well documented. Cancer cell lines including the 5-8F nasopharyngeal carcinoma cells, the HCT116 colorectal cancer cells and the THP-1 acute monocytic leukaemia cells harbor hundreds of miRNAs that are enriched in the nucleus of these cells [28, 29, 58]. The nuclear-specific functions of many of these miRNAs remain elusive, but they are likely to regulate or fine-tune the expression of cancer-associated genes. For example, miR-10a, which is nuclear-localized in both the HCT116 and THP-1 cell lines [29, 58], has been reported to inhibit the transcription of *Hoxd4* in the breast cancer cell lines, MCF7 and MDA-MB-231 [39]. Nuclear-localized miR-10a binds with near perfect complementarity to the promoter of this tumor invasion and metastasis-promoting gene to trigger its silencing via hypermethylation and trimethylation of histone 3 lysine 27 at its promoter [39]. This example demonstrates that nuclear enrichment of miR-10a may provide a therapeutic opportunity to modulate gene expression relevant to cancer metastasis.

Other nuclear-localized miRNAs have been reported to promote transcriptional activation of oncogenes by binding to their promoters. Examples include miR-483 that binds to the promoter of *IGF2* to increase its expression in Wilms’ tumors [59] and miR-558 that binds to the promoter of heparanase (*HPSE*) to enhance its expression, resulting in enhanced tumor growth in neuroblastoma cells [44] (Table 2).

**Table 2:** Function of nuclear-localized miRNAs relevant to human cancers

Nuclear miRNA	Function	Cancer cells	Reference
miR-10a	Inhibit *Hoxd4* to control cancer metastasis	Breast cancer cell lines: MCF7 and MDA-MB-231	[39]
miR-483	Activate *IGF2* to enhance tumorigenesis	Wilms’ tumors	[59]
miR-558	Activate *HPSE* to promote tumor growth	Neuroblastoma cell lines: SK-N-SH, SK-N-AS, SH-SY5Y and SK-N-BE(2)	[44]
miR-373	Increase expression of *CDH1* tumor suppressor gene	Prostate cancer cell line: PC3	[38]
miR-124	Activate tumor suppressor gene, *P27*	Breast and ovarian cancer cell lines: MDA-MB-231, HeyA8, SKOV3.ip1, SKBr3, OVCAR-3, BxPC-3 and L3.6pl, MIA PaCa 2, Panc1, U87, SNB19 and LN229	[60].
miR-205	Activate interleukin tumor suppressor genes, *IL24* and *IL32*	Prostate cancer cell lines: PC3, LNCaP and Du145	[45].
miR-774	Promote transcription of *Ccnb1*	Mouse prostate adenocarcinoma cell line: TRAMP C1	[61]
miR-370	Activate tumor suppressor gene, *P21*	Bladder cancer cell lines: T24 and EJ	[76]
miR-1180	Activate tumor suppressor gene, *P21*	Bladder cancer cell lines: T24 and EJ	[76]
miR-1236	Activate tumor suppressor gene, *P21*	Bladder cancer cell lines: T24 and EJ	[76]
miR-939	Repression of *Bcl-xl* anti-apoptosis gene	Human neuroblastoma cell line: SH-SY5Y	[62]

Nuclear-localized miRNAs can also protect against tumorigenesis by promoting the activation of tumor suppressor genes. For example, miR-373 binds to the promoter of *CDH1* to increase its expression in the prostate cancer cell line, PC3 [38]. miR-124 promotes the activation of *P27*, leading to G1 arrest in myriad breast and ovarian cancer cell lines [60]. miR-205 induces the expression of the interleukin tumor suppressor genes, *IL24* and *IL32*, by targeting specific regions of their promoters [45]. Additional examples are provided in Table 2.

The mechanisms by which binding of miRNAs to gene promoters results in transcriptional activation or silencing have also been described. They include increased or decreased levels of histone modifications associated with gene activation [39, 45, 61], altered RNA Polymerase II activity [38, 61], enhanced recruitment of transcription factors [59] and inhibition of transcription factor binding due to the presence of decoy miRNAs [62].

Besides coding genes, cancer-associated nuclear long non-coding RNAs (lncRNAs), *MALAT-1* and *XIST*, have been reported as a target of miR-9 and miR-210, respectively [63, 64]. The HOTAIR lncRNA, which localizes to both the nucleus and cytoplasm, has also been reported as a target of miR34a in prostate cancer cells [65]. However, it is unknown whether this interaction occurs in the nucleus. The GENCODE Consortium has mapped over 10,000 lncRNAs as putative miRNA targets, many of which are nuclear-enriched [66]. Further experimental validations will no doubt identify more nuclear lncRNAs as miRNA targets that are relevant to cancer.

Surprisingly, no report has previously reviewed the role of nuclear miRNAs in haematological malignancies despite the known roles of miRNAs as oncogenes or tumor suppressor genes [18, 67]. miR-15a and miR-16-1 are established tumor suppressor miRNAs in chronic lymphocytic leukaemia. Their biogenesis is known to be regulated by the nuclear-localized miR-709 in liver cells [41, 67]. Whether or not miR-709 fine-tunes the expression of miR-15a and miR-16 in chronic lymphocytic leukaemia remains to be determined. As described in the previous section of this review, nuclear miR-223 has a key role in normal granulopoiesis. A decrease in miR-223 expression level is associated with CEBPA-mutated acute myeloid leukaemia [68]. Inhibition of the miR-223 target gene, *E2F1*, by CEBPA is pivotal to prevent leukemogenesis that results from E2F1-mediated expression of the oncogene tribble (Trib) 2 gene [68, 69]. As such both miR-223 and CEBPA regulate the expression of E2F1 in myeloid cells under normal physiological condition. Whether aberrant nuclear detention of miR-223 can occur to derepress E2F1 in CEBPA mutant leukaemia demands further investigation.

## Challenges in determining the functions of nuclear miRNAs

The role of nuclear-localized miRNAs has been relatively neglected as evidenced by the extensive literature on miRNAs, including those relevant to hemopoiesis and cancer. Indeed, the majority of studies have focused on the action of miRNAs in post-transcriptional gene regulation. Several reports have considered miRNAs’ role in transcriptional regulation of specific mRNAs only when these miRNAs did not act canonically via 3′ UTR targeting [39, 61]. Others have focused on the transcriptional regulation of specific genes by miRNAs because these genes are known to be transcriptionally regulated by other synthetic or endogenous double-stranded small RNAs [37, 38]. In order to promote more rapid elucidation of the nuclear-associated roles of thousands of miRNAs, studies should specifically seek to identify nuclear miRNAs and their functions, notwithstanding the technical challenges.

Most studies reporting nuclear-localized miRNAs have utilized nuclear and cytoplasmic fractionation methods to determine their localization. Inevitably, the difficulty in obtaining a perfectly pure nuclear fraction has provoked scepticism concerning the accuracy of reported studies. Ensuring the removal of the cytoplasmic fraction prior to collection of the nuclear fraction is crucial and not always possible with some cells. It is also important to perform validations to show the absence of contaminating factors by western blot and RT-qPCR in conjunction with microscopy-based detection of nuclear miRNA labelled with fluorescence probes. These experimental procedures are laborious and often require cell-type specific optimizations to obtain reliable results. This is particularly relevant for commercially available nuclear and cytoplasmic extraction kits typically optimized for use with commonly studied cell lines such as HeLa. In our recent work, we undertook considerable optimization steps beyond the manufacturer’s protocol to obtain good quality nuclear and cytoplasmic fractions from primary cells [43].

Upon identification of nuclear miRNAs, one next logical step is to determine where they bind. Bioinformatic tools have been developed to predict targets of miRNAs within gene promoters or pri-miRNAs [70, 71]. Nearly 800,000 miRNA seed sequences match over 27,000 promoter sequences [71]. miRNAs can also target gene promoters through non-seed related complementarity [45], indicating that miRNA binding to promoter regions may be more widespread than previously thought.

However, similar to the complexity of 3′ UTR targeting by miRNAs, predicted miRNA-promoter/pri-miRNA pairing often did not result in the expected functional changes or occur in a cell-type specific manner [38, 43]. Thus, cause-effect experiments are necessary to confirm that the binding of miRNAs to genomic sequences in the nucleus results in functional consequences.

Experiments designed to reduce the expression of miRNAs that localized specifically to the nucleus are technically challenging. Indeed, our recent report shows the dominant cytoplasmic localization of anti-miR-706 when transfected into myeloid cells [43]. In a study that reported the inhibition of miR-15a and miR-16 processing by miR-709 in mouse L929 liver cells, modest upregulation of miR-15a and miR-16 (<2-fold) was detected in anti-miR-709 transfected cells [41]. It has not been shown whether anti-miR-709 entered the nucleus to inhibit miRNA processing. Thus, a direct role of nuclear miR-709 in controlling the expression of other miRNAs remains elusive.

A recent study has reported the utility of a snoRNA-based vector (snoVector) that allows efficient nuclear retention of RNA molecules processed from this vector [72]. RNA sequences such as lncRNAs, coding mRNAs and precursor miRNAs (pre-miRNAs) can be inserted into the snoVector. They can subsequently be processed into functional RNAs via the endogenous snoRNA processing machinery. RNA molecules expressed using snoVector have been reported to be nuclear-enriched, including those that are typically localized to the cytoplasm [72]. A similar vector (snoMEN) created via manipulation of the human C/D small nuclear RNA HBII-180C has also been reported to target nuclear RNA [73]. It has been utilized to express interfering RNAs that effectively reduced the expression of complementary sequences including nuclear pre-mRNA and pri-miRNA [74, 75]. Thus, it may be possible to use snoVector or SnoMEN to constrain anti-miRNA sequences to the nucleus. This step may facilitate functional characterization following specific and efficient repression of nuclear miRNAs.

Nonetheless, it is important to recognize that miRNAs have been reported to shuttle from the cytoplasm into the nucleus. So, even if repression of specific nuclear miRNAs is achieved, the observed functional loss may be due to the overall depletion of cytoplasmic miRNAs that are inhibited when they enter the nucleus. In such a case, it would be very challenging to discern the specific role of a given nuclear-localized miRNA. Optimal experiment design should exclude changes in cytoplasmic miRNA expression as a contributor to any phenotypic alteration. Alternatively, despite experimental hurdles, it is necessary to distinguish the function of any given nuclear miRNA from its cytoplasmic counterpart.

## Conclusions

The importance of miRNAs in hemopoiesis and cancer through the post-transcriptional regulation of the expression of relevant genes has been well-established. In recent years, non-canonical roles for nuclear-localized miRNAs have been uncovered. While there is evidence that nuclear miRNAs regulate the transcription of specific genes in hemopoietic and cancer cells, these examples are relatively few in the context of the vast published literature on miRNAs in these cell types. In this review, we have described nuclear miRNAs that promote granulopoieis and those that enhance the expression of tumor suppressor genes and oncogenes. The roles of nuclear miRNAs in the vast majority of myeloid and lymphoid cells remain to be determined. Their roles in cancer, including post-transcriptional silencing of tumor suppressor genes, is as yet unreported. Therefore, examination of the functions of these nuclear miRNAs should prove to be a fruitful area for further research.

## Abbreviations

3′ UTR: 3′ untranslated region
Ago: Argonaute
*HPSE*: Heparanase
lncRNAs: Long non-coding RNAs
miRNA: MicroRNA
mRNA Messenger RNA pre-miRNA: Precursor miRNA
pri-miRNA: Primary miRNA
RISC: RNA-induced silencing complex
